# Differential diagnosis of a thyroid mass, Gottron's papules, Calcinosis Cutis and Ptosis on the Saint Mary Magdalen and two depictions of a Madonna and Baby by Bartolomeo Vivarini (1432–1499)

**DOI:** 10.1007/s40618-023-02004-8

**Published:** 2023-01-12

**Authors:** H. Ashrafian

**Affiliations:** Institute of Global Health Innovation, Imperial College London, The Department of Surgery and Cancer, St Mary’s Hospital, 10th Floor Queen Elizabeth the Queen Mother (QEQM) Building, Praed Street, London, W2 1NY UK

**Keywords:** Thyroid, Goiter, Dermatomyositis, Ptosis, Calcinosis, Autoimmunity, Iodine deficiency

The evolution of the Renaissance has offered several insights into Renaissance-era pathology through some of its genius painters. Of these, Bartolomeo Vivarini (1432–1499) has been identified as presenting several cases of goiter in his works, two of which have likely been depictions of the same individual [1], and are celebrated as an ideal example of a Gothic style transitioning into mainstream Renaissance elegance.

**Fig. 1:**
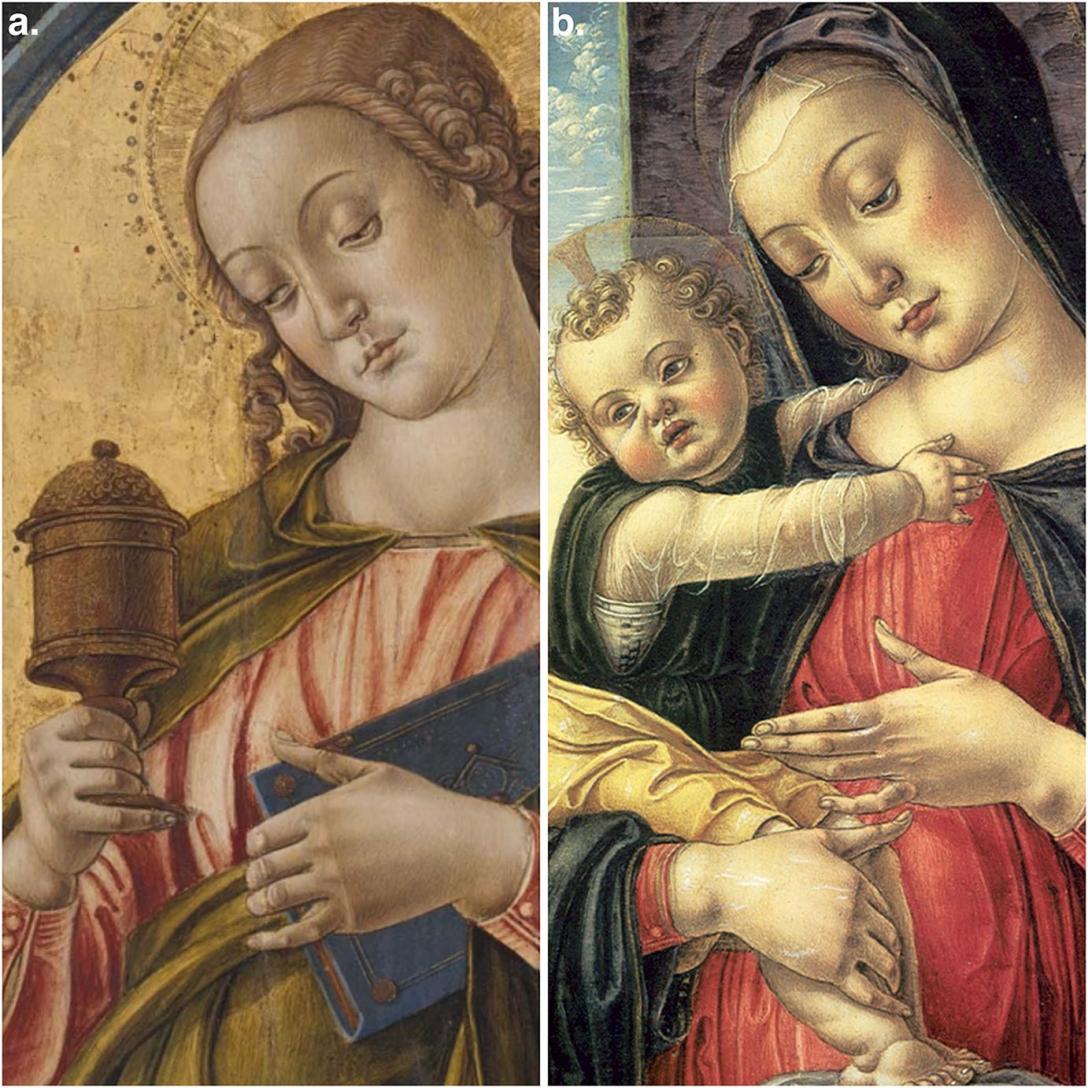
**a** Saint Mary Magdalen (1475) by Bartolomeo Vivarini © Museum of Fine Arts, Boston, USA. **b** Madonna col Bambino (1475) by Bartolomeo Vivarini © National Gallery of Art, Washington D.C., USA

**Fig. 2:**
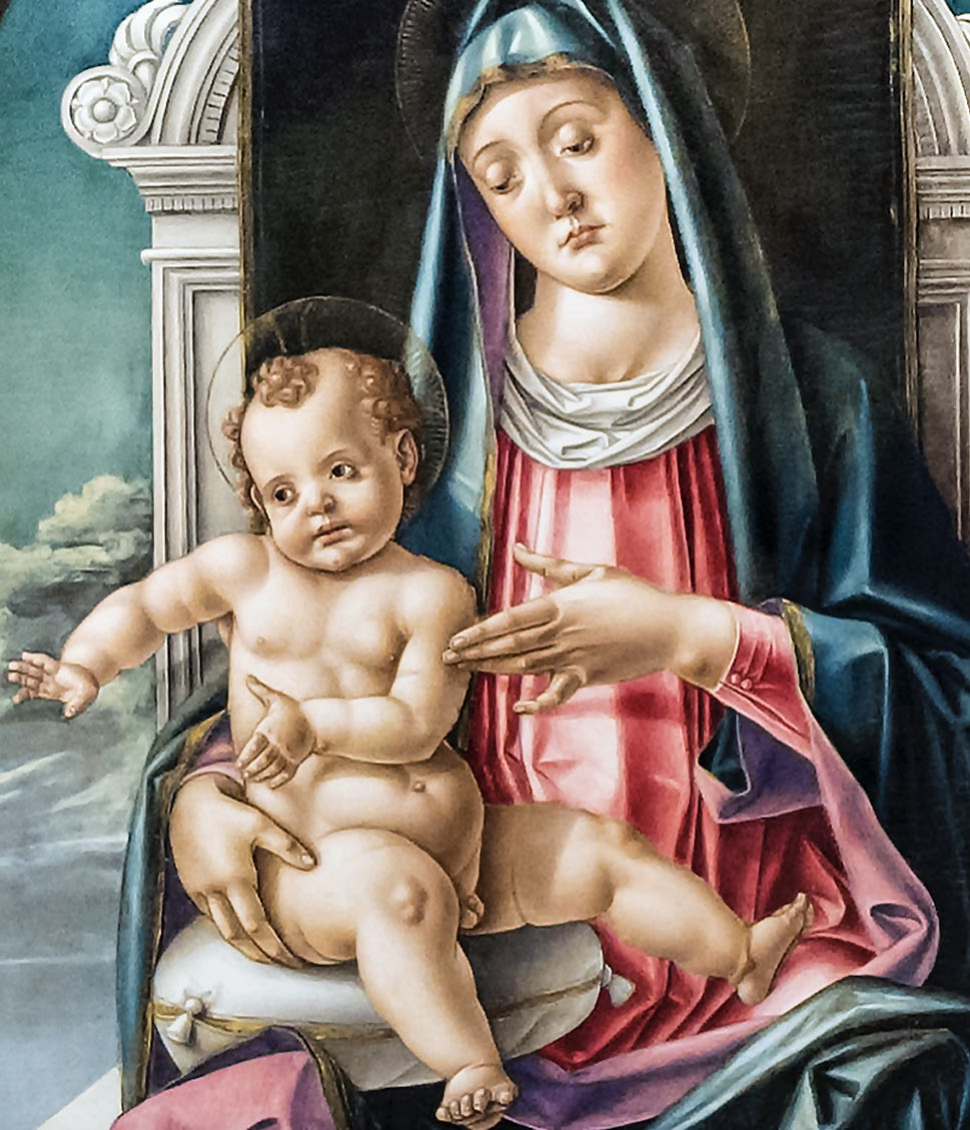
Madonna and Child with Saints (1487) by Bartolomeo Vivarini © Santa Maria Gloriosa dei Frari (the ‘Frari’), Venice, Italy

I note additional pathology and consistency in three Vivarini works. The figure of Saint Mary Magdalen (Fig. 1a) demonstrates a clear neck swelling consistent with a goiter with bilateral ptosis, Gottron's papules at the metacarpophalangeal, proximal interphalangeal and distal interphalangeal joints and Calcinosis Cutis on the ulnar aspect of the right and left distal forearm/wrist. These are directly matching in the Madonna col Bambino (Fig. 1b) and also notable in the Madonna and Child with Saints (Fig. 2) at the Frari in Venice. The clarity of these representations renders the likely possibility that these findings were representative of the source individuals on whom Vivarini based his depictions and quite likely the same individual.

Differential diagnosis of the goiter with ptosis and Gottron’s papules with calcinosis may represent the source individuals depicted may have suffered from concomitant thyroid and dermatopathological autoimmunity. This could be explained by concomitant Hashimoto’s thyroiditis (revealing the goiter and ptosis) and dermatomyositis (Gottron’s papules and calcinosis) [2, 3]. Other autoimmune thyroid conditions such as Graves’ disease (GD) are uncommon in dermatomyositis [2, 3], and whilst dermatomyositis has been associated with an underlying malignancy that can include those of thyroid origin, the thyroid swelling in these cases is much more likely to derive from iodine deficiency or, possibly in this case, autoimmune thyroiditis.

Of note, the figure of the child in the Madonna and Child with Saints (Fig. 2) at the Frari has prominent joint and arm swellings (particularly on the left) that may be artistic in origin, though this might represent calcinosis cutis in the child also, and as a result, possibly the first depiction of juvenile dermatomyositis that may also be connected to the rarity of familial origins for such a condition. In such a case, the goiter would more likely have derived from endemic iodine deficiency.

The likely identification of autoimmunity with goiter has been demonstrated in other renaissance masterpieces [4]. These findings reinforce the possibility of an endemic cause for widespread goiter during the renaissance in Italy whilst also highlighting the exactitude and brilliance of renaissance artists in capturing clinical realities in their artwork and championing the convergence of art and science for a new era.
